# Oral Isotretinoin Resulting in Recurrence of LASIK-Treated Myopia: A Rare Side Effect

**DOI:** 10.7759/cureus.16884

**Published:** 2021-08-04

**Authors:** Khadija Qureshi, Javera Tariq, Maida S Chaudhry, Fajar Pasha

**Affiliations:** 1 Internal Medicine, Knights Medical Associates, Bensalem, USA; 2 Department of Pathology, Division of Hematology, Pakistan Institute of Medical Sciences, Islamabad, PAK; 3 Internal Medicine, DHR Health Institute for Research and Development, Edinburg, USA; 4 Internal Medicine, Holy Family Hospital, Rawalpindi, PAK

**Keywords:** isotretinoin, myopia, adverse effects, acne vulgaris, visual acuity

## Abstract

Isotretinoin is an analog of vitamin A, used to treat severe, recalcitrant nodular acne, psoriasis and disorders of keratinization. However, its benefits come with a broad range of serious side effects, including ocular adverse effects. We report and describe a rare case of bilateral myopia in a female patient treated with oral isotretinoin for acne vulgaris. A female patient, 29-year old, presented to the office for blurry vision. The patient was treated with oral isotretinoin for four weeks. She had a past medical history of myopia precisely corrected with laser-assisted in situ keratomileusis without any residual refractive error. After a thorough examination and laboratory workup, isotretinoin was thought to be the causative agent of her current complaint, so it was immediately stopped. The follow-up showed no further decrement of visual acuity in the patient. However, the vision loss acquired as an adverse effect of oral isotretinoin persisted.

## Introduction

Isotretinoin is a retinoid, a vitamin A derivative, and a powerful drug, approved by the United States Food and Drug Administration in 1982 to treat acne vulgaris and other diseases of hyperkeratinization. Though mainly used to treat nodulocystic acne, it is also used for targeting resistant forms of chronic inflammatory acne. It works by altering DNA transcription leading to decreased size and output of sebaceous glands [1]. It also causes sloughing off of epithelial cells because of its keratolytic action. Isotretinoin has a wide range of mild to severe side effects, including but not limited to dry eyes, keratitis, blepharoconjunctivitis, dry skin, abnormal liver function, nose bleeds, headaches, depressed mood, papilledema, pseudotumor cerebri, central hypothyroidism, inflammatory bowel disease, hepatitis, and pancreatitis. One of the severe and most dreaded side effects of isotretinoin is teratogenicity, potentially causing cleft lip and cleft palate in the fetus. Therefore, it is absolutely contraindicated in pregnancy and is used with caution in women of reproductive age, ensuring proper contraception is done [2]. It is a federally regulated drug that comes with a black box warning. It is only prescribed when a patient’s acne vulgaris fails to respond to other topical and oral treatment methods [1]. Myopia is an ophthalmological condition that causes the visual images to focus in front of the retina rather than on the retina due to elongation of the eyeball. Myopia can be caused by either an abnormal corneal curvature or increased axial length of the eyeball [3]. The refractive angle at which light focuses is distorted, making distant objects blurry. It is also commonly called “nearsightedness” and is measured in diopters (D). Myopia is a prevalent condition, with its prevalence ranging from 30% to 40% in the US and European populations and around 80% in the East Asian population [4]. The causes of myopia are mainly genetic and environmental. It is managed by using corrective glasses or contact lenses, which alter the refractive angle, thus making distant objects appear clear. The surgical treatments are laser-assisted subepithelial keratomileusis, LASIK (laser-assisted in situ keratomileusis), or photorefractive keratectomy. LASIK is the most commonly done procedure and involves using a laser on the cornea to create a thin flap. The cornea is then sculpted with another laser, and the flap is placed back, thus fixing the abnormal corneal curvature. This procedure has an excellent outcome and satisfaction rate without any significant adverse effects. The relatively common and temporary side effects are dry eyes, glare, halos, and double vision, while some of the severe complications of the surgery include astigmatism, flap problems, regression, and over-/under-correction of the refractive error [5].

## Case presentation

**Figure 1:**
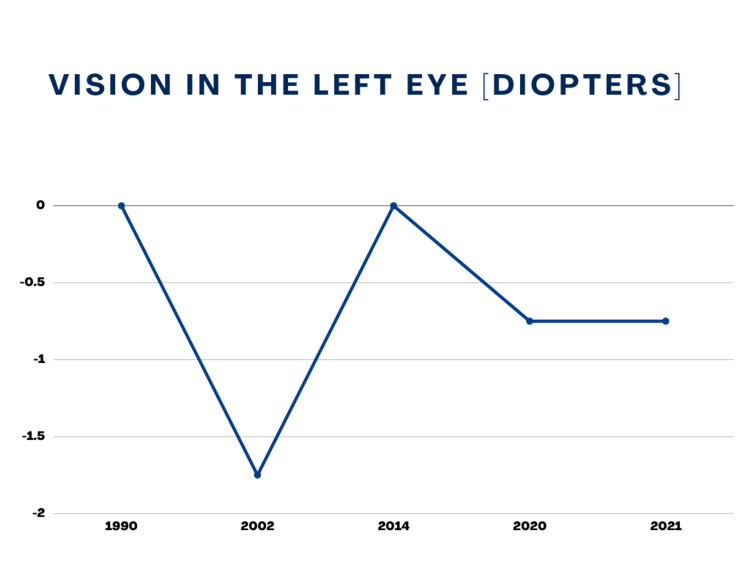
Left Eye Vision (Birth To Date)

**Figure 2:**
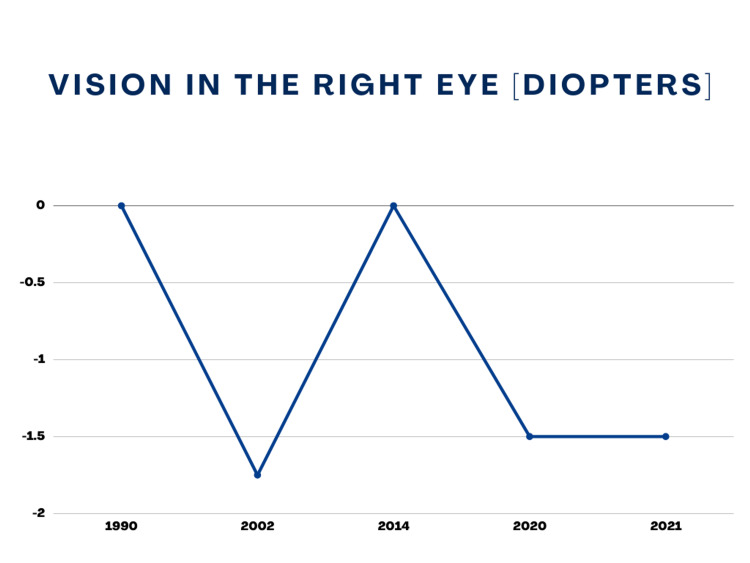
Right Eye Vision (Birth To Date)

A 29-year-old female patient presented to the Internal Medicine Department with the chief complaint of blurry vision for the past month. The patient was a healthy female with a known past medical history of myopia treated with LASIK, and chronic inflammatory acne vulgaris for the past seven years. Apart from that, she had no significant medical or surgical history. Her acne failed to sufficiently respond to topical astringents and keratolytic agents as well as different regimens of topical and oral antibiotics. Four weeks ago, she was prescribed 40 mg or 1.35 mg/kg of oral isotretinoin daily. Before initiating oral retinoid therapy, thorough blood workup including complete blood cell counts, thyroid function test, basic metabolic profile, liver function tests, and lipid panel was performed, revealing no abnormal values. Her annual eye examination also showed visual acuity of 20/20, which is considered the standard visual acuity [6]. The patient had been diagnosed with myopia during early adolescence. At that time, her vision in the right eye was -2.25 D and -1.75 D in the left eye. Her myopia was managed with corrective eyeglasses till she underwent LASIK treatment in 2014. After LASIK in both right and left eyes, her visual acuity became 20/20 bilaterally. She did not experience any side effects of LASIK and her vision stayed stable throughout, as evident by her successive annual eye examinations. Now the patient reported experiencing difficulty in focusing on distant objects. She complained of fuzzy vision as the objects seemed blurry for the first time since undergoing LASIK. She did not exhibit any other signs or symptoms. Her physical examination did not show any abnormalities. Complete laboratory workup was repeated that came out insignificant, showing all the values within normal range. The patient was referred to Ophthalmology, where an optometrist consultation was made. The visual acuity testing showed a vision of -1.50 D in the right eye and -0.75 D in the left eye. The acute recurrence of myopia following oral isotretinoin therapy was considered an adverse effect of the drug. Isotretinoin was immediately discontinued to stop the acutely worsening vision. The patient was prescribed corrective glasses for the refractive error acquired through oral isotretinoin use. For the next six months, follow-up failed to reveal any improvement or worsening in the refractive error in both eyes. Her optometric values over the course of time are shown graphically in Figure 1 and Figure 2. 

## Discussion

Isotretinoin is a potent vitamin A-derived drug, mainly used for the treatment of acne vulgaris as well as other dermatological and hematological diseases like actinic keratosis, folliculitis, rosacea, T-cell lymphoma, neuroblastoma, and prevention of squamous cell carcinoma [1]. It is the only drug showing promising results in permanently reducing resistant forms of acne. Various studies have been conducted regarding its efficacy and relapse rate after 4-6 months of treatment with low to high dosages of oral isotretinoin. Systematic reviews and meta-analysis of a large number of studies show that the success rate with this drug ranges from 85% to 90% in reducing resistant acne and preventing its recurrence in the long term [7]. However, this drug hosts a wide range of side effects, a rare one being new-onset myopia. Intake of Isotretinoin for four weeks daily led to the induction of myopia in our patient whose vision had been previously corrected by LASIK. She did not experience any side effects or complications of LASIK in the interim period, and her vision remained 20/20 throughout until initiating oral isotretinoin for her chronic acne vulgaris. Furthermore, the patient was neither taking any other medication during or around those four weeks of therapy nor had any major lifestyle/dietary changes. Therefore, the introduction of systemic isotretinoin seems to be the only suspicious culprit in this case. Isotretinoin is a powerful drug that acts by modifying DNA transcription [1], leading to an abnormal corneal curvature and an increase in the axial length of the eyeball, generating myopic changes in vision. Thus, isotretinoin has the potential of affecting both pathophysiological mechanisms of myopia [8]. One of the active molecules of isotretinoin is 13-cis-retinoic acid, which is thought to be the main causative ingredient in developing myopia due to this drug. The proposed mechanism by which it affects the cornea is the activation of matrix metalloproteinase 9 activity in the stroma, which causes corneal thinning or melting. That leads to corneal wall weakening and an abnormal curvature causing the myopic refractive error. The remodeling of the corneal layers results in significant differences in surface variance and pachymetry-related parameters of epithelial thickness [9]. Isotretinoin has been vastly studied in the past, and various studies confirm the clear causation of ocular side effects. Several ophthalmic effects caused by this drug have been reported and studied. While many adverse effects are commonly reported with oral retinoids, new-onset myopia has a relatively rare occurrence. A few myopic cases are described in similar patients treated with oral isotretinoin for acne vulgaris. A pilot study carried out in 2020, including 47 patients treated with isotretinoin vs 45 controls, showed isotretinoin to be an important molecule in the etiology of myopia [8]. A similar case was published in 2011 reporting isotretinoin-induced irreversible bilateral high myopia in an Indian patient [10]. In 2017, another case of a 28-year-old female was reported who developed transient myopic shift after two weeks of isotretinoin use. Her medication was stopped immediately, which led to the restoration of normal visual acuity [11]. The American Journal of Ophthalmology has published decreased visual acuity due to isotretinoin as a definite adverse effect after evaluating data from 1745 patients on this drug [12]. Isotretinoin implicates a wide array of more frequently reported ocular side effects, the most common being the meibomian gland degeneration, which manifests as dry eyes, chalazion, blepharitis, and blepharoconjunctivitis [12,13]. Adverse ocular effects that can most certainly be associated with isotretinoin use include abnormal meibomian gland secretion, blepharoconjunctivitis, corneal opacities, decreased dark adaptation, decreased tolerance to contact lens, decreased vision, increased tear osmolarity, keratitis, meibomian gland atrophy, myopia, ocular discomfort, ocular sicca, photophobia, and teratogenic ocular abnormalities. The side effects that can likely be correlated to this drug are decreased color vision and permanent loss of dark adaptation. There is also a possibility of developing permanent keratoconjunctivitis sicca secondary to isotretinoin treatment [11]. The majority of the ophthalmological side effects are diagnosed within four months of starting oral isotretinoin therapy, as evidenced by follow-up studies [13]. Though other side effects of oral isotretinoin have higher incidences and thus are more meticulously studied, myopia induction by isotretinoin is relatively rare. Nevertheless, this particular adverse effect requires vigilant detection and follow-up. Furthermore, it remains to be determined whether the changes in the refractive apparatus of the eye are reversible or permanent damage is done by this drug.

## Conclusions

Isotretinoin carries the risk of inducing myopia or causing progressive worsening of myopia in pre-established myopic cases. Early diagnosis of drug-induced refractive errors can help prevent permanent loss of visual acuity. Cessation of the drug can potentially stop or reverse the vision distortion in such patients, as evident by previous studies. We want to draw the physicians’ attention toward this rare side effect of oral isotretinoin through this case report. Thorough ophthalmological history and testing should be performed on the patients taking oral isotretinoin to avoid this adverse effect. After dispensing the isotretinoin prescription, a follow-up visit to the ophthalmologist should be scheduled. To ensure early detection and treatment, patients and caregivers must be informed of the potential ocular adverse effects of isotretinoin use.
